# Supplementary material for: Patterns of Intron Gain and Loss in Fungi
Source: PLoS Biol. 2004 Nov 30;2(12):e422. doi: 10.1371/journal.pbio.0020422 (PMC532390; doi:10.1371/journal.pbio.0020422)
Supplement: Table S1 — Also available at http://genes.mit.edu/NielsenEtAl/. (4.3 MB ZIP). [file pbio.0020422.st001.zip › NielsenEtAl/html/880.html]

AN2917.1.NCU02840.1.MG01581.1.FG00559.1


```
 CLUSTAL W (1.82) Multiple Sequence Alignments - Introns Inserted


Sequence 1: NCU02840.1	439 aa
Sequence 2: FG00559.1	440 aa
Sequence 3: MG01581.1	439 aa
Sequence 4: AN2917.1	443 aa
Alignment Length: 444 aa
Number Identitical Residues: 399 aa
Alignment Score (without introns) 15323


MG01581.1 	M0PSATGQNWEKYQKNFADDEIEEKKITPLSDE2DIQVLKTYGAAPYGAALKKLEQQIKE
NCU02840.1	M0PSATGQNWEKYRKTFGDDEVEEKKITPLSDE2DIQVLKTYGAAPYGAAIKKLEQQIKE
FG00559.1 	M0PSATGSNWEKYQKNFADDEVEEKKITPLTDE2DIQVLKTYGAAPYGTSIKKLEKQIKE
AN2917.1  	M~PSATGENWEKYKKNFADDEEPEKKITPLTDE2DIAVLKTYGAAPYANALKKLEKQIKE
          	* *****.*****:*.*.***  *******:** ** **********. ::****:****

MG01581.1 	KQQSVDEKIGVK0ESDTGLAPPHLWDVAADRQRMLEEQPLQVARCTKIIQDEK-DESKSK
NCU02840.1	KQQSVDEKIGVK0ESDTGLAPPHLWDVAADRQRMSEEQPLQVARCTKIIPDEK-DESKSK
FG00559.1 	KQQSVDEKIGVK0ESDTGLAPPHLWDIAADRQRMSEEQPFQVARCTKIIADEKGDESKSK
AN2917.1  	RQASVNEKIGVK0ESDTGLAPPHLWDVAADRQRMAEEQPLQVARCTKIIQDEK-DSDKSK
          	:* **:****** *************:******* ****:********* *** *..***

MG01581.1 	YVINVKQIAKFVVQLGERVSPTDIEEG~M----RVGVDRNKYQILLPLPPKIDASVTMMT
NCU02840.1	YVINVKQIAKFVVQLGERVSPTDIEEG~M----RVGVDRNKYQILLPLPPKIDASVTMMT
FG00559.1 	YVINVKQIAKFVVQLGERVSPTDIEEG~M----RVGVDRNKYQIMLPLPPKIDASVTMMT
AN2917.1  	YVINVKQIAKFVVNLGERVSPTDIEED1LSHTDRLSVDRNKYQIMLPLPPKIDPSVTMMT
          	*************:************. :: :.*:.********:********.******

MG01581.1 	VEEKPDVTYGDVGGCKEQVEKLREVVEMPLLSPERFVNLGIDPPKGALLYGPPGTGKTLC
NCU02840.1	VEEKPDVTYGDVGGCKEQVEKLREVVEMPLLSPERFVNLGIDPPKGALLYGPPGTGKTLC
FG00559.1 	VEEKPDVTYGDVGGCKEQVEKLREVVEMPLLSPERFVNLGIDPPKGALLYGPPGTGKTLC
AN2917.1  	VEDKPDVTYGDVGGCKEQIEKLREVVEMPLLSPERFVNLGIDPPKGALLYGPPGTGKTLC
          	**:***************:*****************************************

MG01581.1 	ARAVANRTDATFIRVIGSELVQKYVGEGARMVRELFEMARTKKACIIFFDEIDAVGGARF
NCU02840.1	ARAVANRTDATFIRVIGSELVQKYVGEGARMVRELFEMARTKKACIIFFDEIDAIGGARF
FG00559.1 	ARAVANRTDATFIRVIGSELVQKYVGEGARMVRELFEMARTKKACIIFFDEIDAIGGARF
AN2917.1  	ARAVANRTDATFIRVIGSELVQKYVGEGARMVRELFEMARTKKACIIFFDEIDAVGGARF
          	******************************************************:*****

MG01581.1 	DDGAGGDNEVQRTMLELITQLDGFDARGNIKVMFATNRPSTLDPALMRPGRIDRKIEFSL
NCU02840.1	DDGAGGDNEVQRTMLELITQLDGFDARGNIKVMFATNRPSTLDPALMRPGRIDRKIEFSL
FG00559.1 	DDGAGGDNEVQRTMLELITQLDGFDARGNIKVMFATNRPSTLDPALMRPGRIDRKIEFSL
AN2917.1  	DDGAGGDNEVQRTMLELITQLDGFDARGNIKVMFATNRPSTLDPALMRPGRIDRKIEFSL
          	************************************************************

MG01581.1 	PDLEGRANILRIHAKSMSVERDIRWELISRLCPNATGAELRSVCTEAGMFAIRARRKVAT
NCU02840.1	PDLEGRANILRIHAKSMSVERDIRWELISRLCPNATGAELRSVCTEAGMFAIRARRKVAS
FG00559.1 	PDLEGRANILRIHAKSMSVERDIRWELISRLCPNATGAELRSVCTEAGMYAIRARRKVAS
AN2917.1  	PDVEGRANILRIHAKSMSVERDIRWELISRLCPNATGAELRSVATEAGMFAIRARRKVAT
          	**:****************************************.*****:*********:

MG01581.1 	EKDFLSAVEKVIKGNLKFNSTASYMQYN
NCU02840.1	EKDFLSAVDKVIKGNLKFNSTAAYAQYN
FG00559.1 	EKDFLSAVDKVIKGNLKFNSTATYMQYN
AN2917.1  	EKDFLAAVDKVIKGNLKFNSTATYMQYN
          	*****:**:*************:* ***
```
